# *Cryptosporidium parvum* infections in a cohort of veterinary students in Sweden

**DOI:** 10.1017/S0950268814003318

**Published:** 2015-01-30

**Authors:** P. KINROSS, J. BESER, K. TROELL, C. SILVERLÅS, C. BJÖRKMAN, M. LEBBAD, J. WINIECKA-KRUSNELL, J. LINDH, M. LÖFDAHL

**Affiliations:** 1 Surveillance and Response Support Unit, European Centre for Disease Prevention and Control (ECDC), Sweden; 2 European Programme for Intervention Epidemiology Training (EPIET), ECDC, Sweden; 3 Public Health Agency of Sweden, former Swedish Institute for Infectious Disease Control, Solna, Sweden; 4 National Veterinary Institute (SVA), Uppsala, Sweden; 5 Swedish University of Agricultural Sciences, Uppsala, Sweden (SLU), Sweden

**Keywords:** *Cryptosporidium*, investigation, occupation-related infections, outbreaks, veterinary pathogens

## Abstract

In March 2013, a veterinary student tested positive for *Cryptosporidium*; four classmates reported similar gastrointestinal symptoms. We aimed to identify source(s) and risk factors for *Cryptosporidium* infection in university persons symptomatic between 21 January and 14 April 2013. Sixty-four (79%) students from a cohort of 81 fourth-year veterinary students completed questionnaires, identifying 13 cases; four were *Cryptosporidium parvum* GP60 subtype IIaA16G1R1b, two were IIdA24G1, seven did not submit stool samples. Thirteen cases attended the university's field clinic before symptom onset (13/37 attendees, 35%); 11 visited at least one of four farms where students recalled seeing calves with diarrhoea. *C. parvum* subtype IIaA16G1R1b was identified in calves at one of the farms. Entering pens of calves with diarrhoea [relative risk (RR) 7·6, 95% confidence interval (CI) 1·7–33·5] and eating in clinic cars (RR 9·1, 95% CI 1·3–65·8) were associated with being a case. Washing hands at least twice per farm visit (0 cases, *P* = 0·03) was protective. This outbreak investigation was notable for rapid and effective collaboration between public health, veterinary and environmental sectors, leading to swift identification of a microbiological and epidemiological link between cases, infected calves and their farms. We recommend frequent hand-washing using proper technique and dissuasion from eating in clinic cars to minimize possible exposure to contaminated surfaces.

## INTRODUCTION

*Cryptosporidium* is a protozoal parasite with an environmentally robust oocyst stage that is infectious when excreted. *C. parvum,* one of the major *Cryptosporidium* species in humans, can be associated with zoonotic infection. Calves can be a major reservoir as *C. parvum*-infected calves' manure contains high oocyst concentrations [1, 2]. In Sweden, *C. bovis* is the most common species in calves, but in herds with calf diarrhoea caused by *Cryptosporidium*, >90% of calves are infected with *C. parvum* [3–7]. Other clinically important *Cryptosporidium* species include the anthroponotic species *C. hominis*, often involved in waterborne outbreaks, and the zoonotic species *C. meleagridis, C. felis*, and *C. canis* [8].

The infectious *C. parvum* and *C. hominis* dose for humans is low at ⩾10 oocysts, but might be higher for other *Cryptosporidium* species [9]. The incubation period is usually 2–10 days (average 7 days), and the most common symptom is watery diarrhoea. Other symptoms can include stomach cramps, dehydration, nausea, vomiting, fever, or weight loss. The infection is self-limiting and can be asymptomatic in immunocompetent people, but more severe cases may require hospitalization [10].

Infection can be prevented by reducing people's exposure to contamination (e.g. contaminated food or drinking water), and promoted by reinforcing hand hygiene measures, particularly during and following contact with potentially infected symptomatic people, calves and other animals [11].

While infection in cattle is not notifiable in Sweden, human *Cryptosporidium* infection has been notifiable since 1 July 2004 (Communicable Disease Act; Smittskyddslagen 2004: 168), with diagnosing clinicians and laboratories reporting all cases to the Public Health Agency of Sweden (until December 2013; the Swedish Institute for Communicable Disease Control (Smittskyddsinstitutet; SMI) and the County Medical Officer. In 2012, 238 cases were notified to SMI in Sweden. In the Uppsala region in 2008–2012, fewer than one case was notified each month on average (mean 0·8/month) [12].

Sweden has experienced several food- and waterborne outbreaks of *Cryptosporidium* in the years prior to this outbreak [13-16]. The outbreaks caused by contamination of the municipal drinking water with *C. hominis* in Östersund and Skellefteå in 2010 and 2011, were notable due to the large number of infected people (total 46000) [17].

### Veterinary studies and ambulatory clinic classes at the Swedish University of Agricultural Sciences

In 2013, 360 students at the Swedish University of Agricultural Sciences (Sveriges lantbruksuniversitet; SLU) in Uppsala, Sweden were veterinary students, of which 81 were in their fourth year, attending class between 21 January and 13 December 2013. Ten classes were taught on the SLU campus, while one class, coordinated by the animal hospital's ambulatory clinic, was taught off campus. The ambulatory clinic is an on-call centre with 10 staff. Its veterinarians triage phone calls at SLU each morning, then drive groups of students to farms in ambulance cars. This provides students with their first opportunity for intense, hands-on clinical education on farms, e.g. examining animals with diarrhoea. Veterinary students only have classes there in their fourth year spring and fifth year autumn semesters. By 29 March 2013, 40 fourth-year students had attended ambulatory clinic classes, visiting more than 50 different farms with animals including cattle, horses and sheep.

### Initial alert and response

On 9 February 2013, a fourth-year veterinary student at SLU experienced the onset of diarrhoea and stomach cramps, sought healthcare, and tested positive for *Cryptosporidium* infection. On 5 March the student reported to the head of the ambulatory clinic that classmates were reporting similar symptoms. All had attended classes at the veterinary hospital's ambulatory clinic on the SLU campus.

SLU immediately initiated internal investigations to confirm the outbreak, and contacted SMI on 7 March. An investigation was initiated by an outbreak control team (OCT) comprising representatives from SMI, the National Veterinary Institute (Statens Veterinärmedicinska Anstalt; SVA) and SLU. Its aims were to describe the outbreak's size, identify potential infection source(s), and identify risk factors for infection to help prevent further cases and similar outbreaks in similar settings.

## METHODS

### Case-finding and hypothesis generation

During initial investigations and case-finding, a case was defined as a veterinary student or staff member at SLU with symptoms of diarrhoea (>2 loose stools within 24 h) with onset after 1 January 2013. On 5 March SLU initiated enquiries with SLU staff and fourth-year students to identify those with cryptosporidiosis-compatible symptoms. SLU contacted all fourth-year veterinary students on 6 March.

SLU conducted unstructured telephone interviews with potential cases to identify common exposures, and reviewed ambulatory clinic records to record their farm visits. On 11 and 12 March SMI made a site visit to SLU to identify plausible risk factors for infection. On 12 March the OCT sent a document to all fourth-year students describing the outbreak, reinforcing standard control measures (including hand hygiene and hygiene measures for cattle farm visits), and directing symptomatic students to contact healthcare and SLU. On 12 March, SLU also searched for cases in other student years by re-contacting all SLU staff who taught clinical students.

Additional cases were sought in the electronic national surveillance system (SmiNet) by searching for cases reported between 1 December 2012 and 30 April 2013 in Uppsala County.

### Calf faecal samples

Faecal samples were collected by SLU's ambulatory clinic staff from five calves aged <6 weeks from cattle herds at two farms suggested by hypothesis-generating interviews in April 2013. If present, calves with diarrhoea were sampled preferentially.

### Microbiological investigations

Symptomatic students' faecal specimens were sent to SMI for detection of *Cryptosporidium* oocysts by modified Ziehl–Neelsen staining. Additional stool parasites were investigated by light microscopy after formol/ethyl acetate concentration. The presence of *Giardia* cysts was analysed using a direct immunofluorescent test (Agua-Glo, Waterborne Inc., USA). DNA was extracted directly from stool specimens using the QIAamp DNA mini kit (Qiagen, Germany) according to the manufacturer's recommendations. Disruption of oocysts was performed before extraction using a BulletBlender (Techtum, Sweden). Species identification was performed by amplification of the small subunit rRNA (SSU rRNA) gene followed by restriction fragment length polymorphism analysis [18, 19]. Subtyping was achieved by sequencing an amplified fragment of the 60-kDa glycoprotein (GP60) gene [20].

SVA performed microscopic analysis, SSU rRNA and GP60 typing of calf faecal samples. Molecular analysis of SSU rRNA was performed using Sanger sequencing [19]. Subtyping of calf samples were performed using the same laboratory protocol as on the human samples.

### Epidemiological cohort study

The OCT initiated a cohort study to identify risk factors for symptomatic *Cryptosporidium* infection among fourth-year SLU veterinary students in January–April 2013, and to inform recommendations to prevent further cases.

For this epidemiological investigation, a probable case was defined as a veterinary student at SLU with diarrhoea (>2 loose stools within 24 h) between 21 January (the start of the semester) and 5 April 2013; a confirmed case was a probable case with laboratory-confirmed *Cryptosporidium* infection; cases were excluded if their stool specimens were negative for *Cryptosporidium*.

An online questionnaire was generated using Survey Generator™ (http://www.alstra.se/en/survey-tool), and sent electronically to the cohort on 15 March. The questionnaire collected data on cryptosporidiosis symptoms experienced since 25 December 2012 (i.e. >2 incubation periods prior to the semester, to permit a reliable description of the outbreak's start), SLU-centred factors (e.g. contact with the ambulatory clinic, farms and symptomatic animals; lunch in cars), and non-SLU-centred factors (e.g. close contact with cases outside SLU, non-SLU animal contact). A list of farms visited by cases was provided by SLU (i.e. farms A–P); respondents were asked which farm they visited. Students who reported symptoms after questionnaire completion were sent a new questionnaire, and their original response was discarded.

Descriptive analyses examined cases' clinical, geospatial and temporal characteristics, and common exposures.

Cohort data analyses estimated associations between being a case and potential risk factors using *χ*^2^ and Fisher's exact tests if there were fewer than five exposed or unexposed cases or non-cases, or fewer than 20 respondents to a question. Ordered categorical variables with few cases per category were recoded as binary variables following univariate analysis and/or logistic regression. Test for trend for ‘weeks spent in the ambulatory clinic’ was analysed assuming a categorical variable using a non-parametric test, and by univariate logistic regression.

Multivariable analyses by logistic regression of variables which had evidence of an association by univariate analysis are not presented, as no statistically significant associations were identified due to insufficient statistical power. All analyses were performed in Stata/SE v. 12.0 (StataCorp LP, USA) and Microsoft Excel 2010 (Microsoft Corp., USA).

## RESULTS

### Hypothesis generation

Hypothesis-generating interviews with eight cases identified that all had attended class at the ambulatory clinic in 2013. During that class, they visited 16 different farms, 4–6 each. All had visited farms A and/or B. Clinic staff recalled that these two farms had calves with diarrhoea in 2013. Clinic staff did not know of any clinic co-workers, or farm workers who had experienced cryptosporidiosis-compatible symptoms in the previous 6 months.

The hypothesis that infection of these students followed contact with the ambulatory clinic and/or farm A and/or farm B in 2013 was found to be plausible during the March site visit. Therefore the OCT recommended that calf faecal specimens be taken from farms A and B, and initiated an analytical study to test the hypothesis.

### Environmental investigations

There were no cattle with diarrhoea at farm A when faecal specimens were collected, while there were calves with diarrhoea at farm B during collection.

### Microbiological investigations

*Cryptosporidium* infection was confirmed by microscopy and polymerase chain reaction in 6/7 faecal specimens submitted for analysis. No other stool parasites were found by extended microscopy; anti-*Giardia* monoclonal antibody was negative for the presence of *Giardia* cysts in all eight specimens. Molecular analysis identified *C. parvum* subtype IIaA16G1R1b (100% identical to EU647728) and *C. parvum* subtype IIdA24G1 (100% identical to HQ005751) in five and two specimens, respectively.

*C. parvum* subtype IIaA16G1R1b was identified in calf faecal specimens that were taken in April 2013 from farm B, but no *Cryptosporidium* oocysts could be found in specimens from farm A.

### Epidemiological investigations

The cohort of 81 fourth-year students returned 69 questionnaire responses (response rate 84%); 91% of the respondents were female, representing the demographics of fourth-year students (~80% female). Four responses were excluded from analysis (one met the exclusion criteria; three did not indicate symptom onset dates, essential data for classification of cohort members as cases or non-cases).

The cases definition was met by the questionnaire responses of 13 students, therefore the attack rate (AR) in the cohort was 20% (13/65 students). Six of these were confirmed cases, and seven were probable cases. Their mean age was 26 years with all except one being female.

**Fig. 1.**
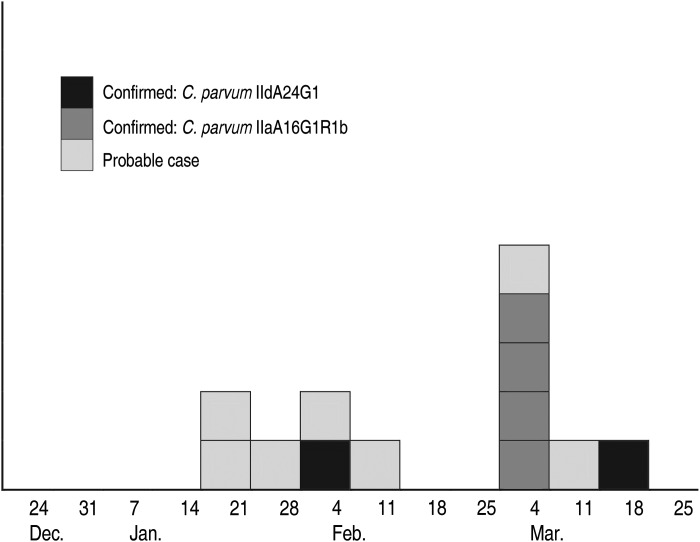
Cases of cryptosporidiosis by subtype and week of symptom onset between 24 December 2012 and 31 March 2013 (*n* = 13)

The cases' symptom onset dates were between 24 January and 18 March 2013, i.e. none prior to the 2013 spring semester (Fig. 1). Ten cases reported watery diarrhoea for at least 1–2 days (71%). Two cases sought medical care, one of whom was admitted to hospital for a day. Ten (71%) cases took at least a day off class due to this illness (mean 4 days, maximum 10 days, sum of all missed days = 52 days).

The ambulatory clinic had been attended by all 13 cases identified in the cohort study, of which 12 attended clinic classes; the thirteenth case attended the clinic for just 1 day. Cases had used all five clinic cars; all but one had eaten lunch or snacks in the cars. Cases reported seeing animals with diarrhoea at farms A, B, C and D only (Table 1). The only animals reported with diarrhoea were ‘cows’ or ‘calves’. Eleven of 13 cases visited at least one of these farms (85%, Table 2).

**Table 1.** Farms with animals with diarrhoea visited by cases in 2013 between 21 January and 31 March 2013

Farm	Number of cases who reported visiting farm	Cases who recall seeing animals with diarrhoea at that farm
Farm A	9	3
Farm B	5	2
Farm C	3	2
Farm D	2	1
Other farms (*n* = 12 farms)	13	0

**Table 2.** Univariate analysis of risk factors in a cohort of fourth-year veterinary students for an association with possible symptomatic cryptosporidiosis infection (n = 65)

	Exposed			Unexposed			RR		
	Total	Cases	AR%	Total	Cases	AR%		95% CI	*P* value
Male gender	6	1	17	59	12	20	0·82	(0·13–5·26)	1
Older than median (26·4 years)	33	4	12	32	9	28	0·43	(0·15–1·26)	0·13
Visited at least one of farms A, B, C or D	38	11	29	12	2	17	1·67	(0·45–6·76)	0·48
Visited any farm	50	13	26	15	0	0	–	(–)	0·029
Farm A	36	9	25	14	4	29	0·88	(0·32–2·39)	1·0
Farm B	7	5	71	43	8	19	3·84	(1·76–8·39)	0·009
Farm C	6	3	50	44	10	23	2·20	(0·84–5·79)	0·17
Farm D	4	2	50	46	11	24	2·09	(0·69–6·33)	0·28
Farm E	4	2	50	46	11	24	2·09	(0·69–6·33)	0·28
Farm F	1	0	0	49	13	27	0·00	(–)	1
Farm G	4	3	75	46	10	22	3·45	(1·57–7·59)	0·049
Farm H	4	1	25	46	12	26	0·96	(0·16–5·60)	1
Farm I	2	2	100	48	11	23	4·36	(2·60–7·33)	0·064
Farm J	1	1	100	49	12	24	4·08	(2·50–6·68)	0·26
Farm K	8	3	38	42	10	24	1·58	(0·55–4·48)	0·41
Farm L	4	1	25	46	12	26	0·96	(0·16–5·60)	1
Farm M	0	0	.	50	13	26	–	(–)	.
Farm N	6	3	50	44	10	23	2·20	(0·84–5·79)	0·17
Farm O	1	1	100	49	12	24	4·08	(2·50–6·68)	0·26
Farm P	1	1	100	49	12	24	4·08	(2·50–6·68)	0·26
Other farm(s)	25	5	20	25	8	32	0·63	(0·24–1·65)	0·52
Spent at least 1 day at ambulatory clinic	37	13	35	28	0	0	–	(–)	0
Had a week-long class in ambulatory clinic	34	12	35	31	1	3	10·94	(1·51–79·33)	0·001
Entered building that held calves with diarrhoea	23	9	39	31	2	6	6·07	(1·45–25·45)	0·005
Entered a pen that held calves with diarrhoea	15	6	40	38	2	5	7·60	(1·72–33·54)	0·004
Hands-on contact with a calf with diarrhoea	19	7	37	39	3	8	4·79	(1·39–16·49)	0·010
Hands-on contact with any other animal with diarrhoea	23	4	17	30	4	13	1·30	(0·36–4·67)	0·7
Ate in a clinic car (lunch and/or snacks)	37	12	32	28	1	4	9·08	(1·25–65·77)	0·004
Wore protective gloves in clinic cars at least once	8	1	13	39	12	31	0·41	(0·06–2·70)	0·4
Used additional hand sanitizers at least once	39	6	15	16	6	38	0·41	(0·16–1·08)	0·071*
Usually use additional hand gel	18	1	6	37	11	30	0·19	(0·03–1·34)	0·078
Pairs of gloves per cattle farm visit (2–3 *vs.* >3)	17	6	35	26	7	27	1·31	(0·53–3·23)	0·6*
Wash hands >1 time per cattle farm visit	22	4	18	22	9	41	0·44	(0·16–1·23)	0·19
Wash hands >2 times per cattle farm visit	9	0	0	35	13	37	0·00	(–)	0·04
Drank tap water on farm	4	3	75	58	10	17	4·35	(1·96–9·67)	0·03
Smokes, even occasionally	2	0	0	61	13	21·31	0·00	(–)	1
Takes moist snuff ('snus'), even occasionally	1	0	0	63	13	20·63	0·00	(–)	1
Lives with someone who has professional animal contact	10	3	30	54	10	18·52	1·62	(0·54–4·87)	0·41
Lives on or next to property with non-pet animals	4	1	25	59	12	20·34	1·23	(0·21–7·22)	1
Prior to 2013, lived on a cattle farm	12	2	16·67	52	11	21·15	0·79	(0·20–3·10)	1

AR, Attack rate; RR, relative risk; CI, confidence interval.

* *P* value calculated using *χ*^2^ test, all other *P* values calculated using Fisher's exact test.

Cohort study data analysis identified 13 risk factors with strong evidence of an association with being a case (*P*⩽0·05), and six others with some evidence (*P*⩽0·125) (Table 2).

The strongest association was ambulatory clinic attendance [relative risk (RR) 10·9, 95% confidence interval (CI) 1·5–79·3]. The AR for students who spent a day or more in the ambulatory clinic was 35% (13/37), and higher in those attending 4 weeks of class (7/19 students, AR 37%) than 1 week (5/15 students, AR 33%) (*P* = 0·02).

The next strongest associations were eating in cars (RR 9·1, 95% CI 1·3–65·8), and drinking tap water on farms (RR 4·4, 95% CI 2·0–9·7). All cases reported washing their hands fewer than twice per visit to a cattle farm, whereas 13/37 non-cases (AR 37%) reported hand-washing at least twice per visit, providing strong evidence for it being protective (*P* = 0·03).

There was strong evidence in the cohort data of a risk associated with visits to farm B (*P* = 0·009), and also farms G and I (*P* < 0·05), but no statistically significant risk associated with visits to farms A, C or D, nor from visiting ‘at least one of farms A, B, C or D’, i.e. the four farms on which students recalled seeing animals (calves) with diarrhoea (0·2 < *P* < 1·0, Table 2).

The univariate cohort analysis was restricted to the subset reporting ambulatory clinic attendance. All variables were tested, and two factors retained evidence of association with being a case (*P* < 0·1): drinking tap water on a farm (RR 3·3, 95% CI 2·0–5·5, *P* = 0·04), and attending farm B (RR 1·2, 95% CI 1·2–5·5, *P* = 0·073).

**Fig. 2.**
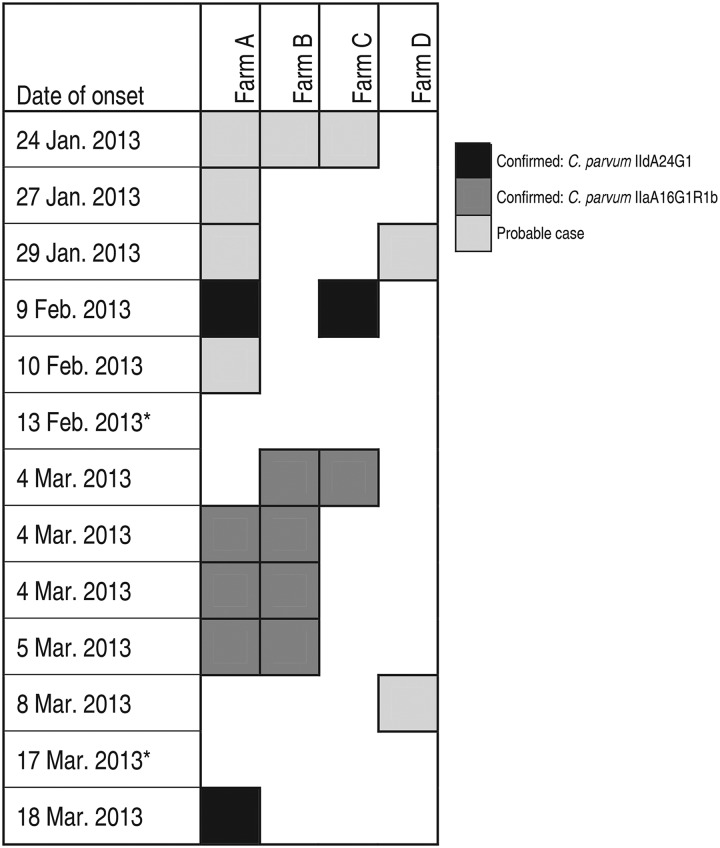
Farms visited by cases in the 10 days prior to the onset of symptoms between 21 January 2013 and 31 March 2013 (*n* = 13 cases). * Cases who did not visit farms A, B, C or D.

Four confirmed cases visited Farm B on 1 day in the week commencing 25 February 2013, during their only week of ambulatory clinic classes. Students only used one car that day. They recalled entering a building with calves with diarrhoea and high-risk activities for zoonotic infections, such as de-horning and neutering calves. Their symptom onsets were all during the subsequent week. *C. parvum* IIaA16G1R1b was identified from these four students' faecal samples, and from environmental calf faecal specimens at farm B (Figs 1 and 2).

## DISCUSSION

This investigation benefited greatly from the rapid availability of microbiological results from farms and students during the collaborative, intersectoral investigation. Microbiological subtype analysis is often not available during outbreak investigations; the university's initial investigations were swift and there was a high response rate from the veterinary students to an electronic questionnaire. Together these microbiological and epidemiological data provided evidence of a link between *C. parvum* infections that affected cattle, and students having contact with the animals' diarrhoea.

The transmission of *C. parvum* infection in those students who attended farms with calves with diarrhoea is likely to have been associated with direct animal contact. Those without this direct contact were most likely infected via contaminated objects in the working environment, e.g. on the farms themselves, in the veterinary hospital's ambulatory clinic, and/or while eating within the ambulatory clinic's cars.

Zoonotic outbreaks of *C. parvum* infection in veterinary students are well documented, and commonly associated with calf contact concomitant with lapses in hygiene measures [1, 2, 10, 21-23]. Investigation of one of these outbreaks was also initiated by a concerned veterinary student together with their university [22]. In 2003, a *C. parvum* outbreak in 68 students in Minnesota related to infected calves had two equally sized epidemic waves, in two sequential school semesters. The recurrence was attributed to inadequate implementation of recommended control measures for barrier protection and hand hygiene [23]. Subsequent to our investigation into this current outbreak, *Cryptosporidium* infection was diagnosed in a fourth-year veterinary student in early May 2013, and another student in July 2013, i.e. after the initial control measures were recommended and SMI's 19 April 2013 outbreak report. This may indicate that the implemented control measures were not sufficient, transmission outside of SLU, or a missed risk factor.

Most of the identified risk factors were plausible. Students who attend ambulatory clinic classes will attend cattle farms, potentially high-risk locations for zoonotic *C. parvum* infection if the calves have diarrhoea [24]. Clinic attendance necessarily includes intense hands-on clinical work on farm animals including calves. Indeed cases reported procedures likely to have resulted in risky contact with infectious material, and so there was potential exposure to high oocyst burdens. Eating in clinic cars was a near ubiquitous experience for clinic attendees, also presenting a potential site for oocyst ingestion. Students were usually out all day, typically eating lunch in the cars. Uniform protective clothing is provided to students by SLU, washed following each day's farm visits with detergent at 40 °C and hung to dry. On the next day it is stored in boxes between trips. Perhaps this protocol provides a possible site for distribution of oocysts; washing this clothing at 40 °C may be insufficient to inactivate oocysts [11, 25].

One notable finding was the epidemiological evidence for consumption of farms' tap water being a risk factor. Indeed the animal facilities at farm A have their own water supply, and farms B, C and D also have their own supplies. As no environmental samples were taken from water supplies on any farm visited by cases there are no direct data to exclude these supplies as a source for infections. However, only three cases reported consuming farm tap water, and there were no notifications in SmiNet relating to farm workers in the Uppsala region. In hindsight, we should have taken environmental samples and would have included questions in the cohort study's questionnaire on the tap's location, and how tap water was consumed (e.g. using a container, using hands or drunk from directly). However, the published literature and the data we collected all suggest that veterinary students' direct and/or indirect contact with infected calves' diarrhoea is the more plausible hypothesis in this outbreak.

Other identified risk factors also require more interpretation. Hand sanitizer gels do not kill oocysts, yet there was some evidence that non-cases were more likely to use it than cases. Perhaps this is a reflection of these students’ inclination towards more rigorous hygiene practices generally. Moreover, there was some statistical evidence that those attending farms G, I, J, O and P were more likely to be cases, but these exposures explain three or fewer cases each (Table 2). In terms of impact, few students sought healthcare due to this outbreak, but 52 days of study were missed by the 81 fourth-year students.

The identification of two subtypes may indicate mixed infection on one farm, or two infections on two or more farms. *C. parvum* subtype IIaA16G1R1b (EU647728) is fairly common in Sweden; it was identified in 16% of subtyped *C. parvum* cases in the Stockholm area between 2006 and 2008, including a possible foodborne outbreak [14], and in several sporadic cryptosporidiosis cases in 2012–2013 (SMI, unpublished data). It was identified as the most prevalent subtype in a study conducted in Swedish herds with calf diarrhoeal problems between 2010–2012, identified in 33% (26/79) of the herds and 34% of calves with determined *C. parvum* subtype [4, 5]. Therefore the strong epidemiological evidence for transmission of this subtype at farm B does not exclude the possibility that other herds were a source. Use of sequence-based methods with higher discriminatory power, such as a multi-locus sequence typing scheme for several micro and mini satellites, may have been able to provide stronger evidence of a microbiological link.

*C. parvum* subtype IIdA24G1 (HQ005751) has been rarely identified in Sweden and elsewhere. It was identified as the aetiological agent in a foodborne outbreak in two Swedish cities in 2010 [16]. Otherwise it has only been found twice in Sweden before this outbreak, in sporadic cases in 2011 and 2013 (J. Beser, unpublished data). Subtype IIdA24G1c, which is highly homologous to IIdA24G1, has been identified in four calves from two herds in Sweden [4].

There was epidemiological evidence for subtype IIdA24G1 at farm A, but it was not identified at either farm tested in this study. Farm A had been sampled for presence of *Cryptosporidium* for research purposes twice in 2012, i.e. prior to this outbreak; only *C. bovis* was identified. This is not unusual: >95% herds in Sweden have calves positive for *C. bovis*, with 30–40% calves <10 weeks infected, while *C. parvum* is rarely identified from non-diarrhoeal calves. Therefore, with three repeated samplings negative for *C. parvum*, it is unlikely that this *Cryptosporidium* species was present at this farm.

Factors inherent to the study's design and execution will have affected the obtained results. The cohort was relatively small, providing too few people per category to generate strong statistical evidence from multivariable analyses to control any confounding and detect possible effect modification. The questionnaire elucidated catalogued each case's symptoms; five probable cases experienced watery diarrhoea for more than a day, no probable case reported blood in stool. Even though these are consistent with cryptosporidiosis, no stool sample was available to confirm the categorization. Case-finding was strong in the fourth-year group, but far weaker in other year groups, and so cases may have remained unidentified by the OCT. The definition of a probable case was non-specific to cryptosporidiosis and diarrhoeal symptoms may have been due to other causes. Plausibly, the infections with the common subtype may have occurred on another farm, from a contaminated working environment or person-to-person. Indeed, students can move more or less freely throughout teaching areas, including seminar rooms opposite the ambulatory clinic. The data collected suggest that person-to-person transmission was unlikely (other than indirect transmission in clinic cars or at the ambulatory clinic) as there were no symptomatic persons in the cases’ households or in student peers who did not attend the ambulatory clinic. This investigation focused on identifying the source of infectious *Cryptosporidium* oocysts rather than mapping their distributions. For this reason specimens were taken from calves and not from ambulatory clinic cars (even though soiling was identified in these cars during the site visit), and no environmental testing was performed on protective clothing or the clinic environment.

A current study at SMI invites all laboratories in Sweden to submit stool samples from human *Cryptosporidium* cases for species differentiation and subtyping. *Cryptosporidium* species and subtype identification in calf diarrhoeal samples are currently being performed at SLU. Such projects should permit discussion of the place of the IIaA16G1R1b and IIdA24G1 subtypes within the Swedish *Cryptosporidium* landscape within the next few years.

The identified risk factors enable us to propose evidence-based recommendations for universities to prevent *C. parvum* infections in veterinary students. We recommend that veterinary schools (*a*) continue to reinforce information to students on risk factors for *Cryptosporidium* infection, especially the risks from calves with diarrhoea; and on hygiene recommendations, particularly related to eating and drinking during clinical studies; (*b*) consider recommending that students do not eat in transport cars; and (*c*) ensure that current routines for washing protective clothes and vehicles are sufficient to kill *Cryptosporidium* oocysts.

As *C. parvum* can transmit person-to-person and result in absenteeism in students, healthcare professionals, particularly at primary care, should be sensitized to test for *Cryptosporidium* infection in veterinary students presenting with gastrointestinal symptoms. This would permit early identification of *C. parvum* outbreaks and targeted interventions to end transmission.
